# Metformin Attenuates Behavioral Deficits and Oxidative Stress in a Preclinical Model of Autism

**DOI:** 10.1002/dneu.70061

**Published:** 2026-09-17

**Authors:** Madna Costa Freitas, Renê Felipe de Freitas, Iara Kessila Milhome Vasconcelos, Isadora Porto de Andrade, Maria Helena da Silva Pitombeira, David Ribeiro Fontenele, Pedro Otávio de Freitas Alves, Victoria Soares Diógenes, Ana Maria Sampaio Assreuy, Gislei Frota Aragão

**Affiliations:** ^1^ Superior Institute of Biomedical Sciences State University of Ceará (UECE) Fortaleza Ceará Brazil

**Keywords:** acetylcholinesterase, autism spectrum disorder, metformin, oxidative stress, valproic acid model

## Abstract

Autism spectrum disorder (ASD) is a complex neurodevelopmental condition lacking effective pharmacological treatments. Metformin has emerged as a candidate for drug repurposing in ASD due to its metabolic and neurobiological actions. This study evaluated the in vivo effects of metformin (400 mg/kg, orally) administered from postnatal day 21 to 35 (PND21–35) in male and female offspring of mice prenatally exposed to valproic acid (VPA; 450 mg/kg, i.p.) on gestational day 12.5 (GD12.5). Behavioral tests included the open field, elevated plus maze, Y‐maze, three‐chamber social interaction, and self‐grooming. The prefrontal cortex (PFC) and hippocampus (HPC) were analyzed for oxidative stress markers (malondialdehyde [MDA], reduced glutathione [GSH]), total protein content, and acetylcholinesterase (AChE) activity. In vivo, metformin attenuated several VPA‐associated behavioral alterations, including changes in locomotor/exploratory activity and repetitive behavior, with additional effects on anxiety‐like behavior, sociability, and spatial working memory that varied according to sex and behavioral parameter. Biochemical analyses showed metformin‐associated changes in GSH and MDA that varied across sexes and brain regions. VPA‐induced increases in PFC AChE activity were attenuated by metformin in both sexes. Total protein content also varied according to sex and brain region. These findings provide preclinical evidence that metformin can attenuate behavioral alterations associated with prenatal VPA exposure and modulate oxidative stress‐related markers, with patterns varying across sexes and brain regions.

## 1 Introduction

Autism spectrum disorder (ASD) is a complex neurodevelopmental condition of multifactorial etiology that manifests during early childhood. It is characterized by deficits in social communication and interaction, along with restricted and repetitive patterns of behavior, interests, and activities (American Psychiatric Association 2022). These manifestations reflect disruptions across multiple domains of brain function and suggest widespread neurobiological dysregulation.

Structural and functional alterations in brain regions involved in social, cognitive, and emotional processing, including the prefrontal cortex (PFC) and hippocampus (HPC), have been reported in ASD (Courchesne 2004; Hazlett et al. 2005; Carper and Courchesne 2005; Ecker 2017). Alterations in neural connectivity and neurotransmitter systems have also been described, supporting the involvement of multiple neural circuits in ASD‐related behavioral phenotypes (DiCarlo and Wallace 2022). In parallel, oxidative imbalance has been implicated in ASD pathophysiology, with evidence of increased lipid peroxidation and impaired antioxidant defenses, including alterations in glutathione homeostasis (Kuźniar‐Pałka 2025). Alterations in cholinergic signaling, including changes in acetylcholinesterase (AChE) activity, have also been reported in experimental models, suggesting that both redox and cholinergic processes may contribute to neurobehavioral alterations (Eissa et al. 2020).

According to the Centers for Disease Control and Prevention (CDC), the prevalence of ASD in the United States is estimated at 1 in 31 children aged 8 years, corresponding to approximately 3.2% of that population (Shaw et al. 2025). These data underscore the pressing need for research focused on understanding the underlying mechanisms of ASD and identifying effective therapeutic strategies.

Despite substantial advances, the etiology of ASD remains only partially understood and involves complex interactions between genetic and environmental factors during neurodevelopment (Hallmayer et al. 2011; Genovese and Butler 2023). Among environmental risk factors, prenatal exposure to valproic acid (VPA), an antiepileptic drug, has been associated with an increased risk of neurodevelopmental alterations, including ASD (Christensen et al. 2013; Guerra et al. 2023). Prenatal VPA exposure in rodents produces behavioral and neurobiological alterations relevant to ASD and has therefore been extensively used as a preclinical model (Nicolini and Fahnestock 2018). Importantly, VPA‐induced phenotypes may differ between males and females, highlighting the relevance of including both sexes when evaluating potential therapeutic interventions (Scheggi et al. 2020).

Current ASD management relies largely on behavioral and educational interventions, whereas pharmacological approaches are primarily used to manage associated behavioral symptoms and co‐occurring conditions. Antipsychotic medications may be prescribed for symptoms such as irritability and aggression, but their use can be limited by adverse effects, including weight gain, sedation, and extrapyramidal symptoms (Aishworiya et al. 2024). These limitations have encouraged the investigation of alternative pharmacological strategies targeting biological processes associated with ASD.

In this context, metformin, a biguanide widely used for the treatment of Type 2 diabetes, has attracted interest as a candidate for drug repurposing in neurodevelopmental disorders. Beyond its metabolic effects, metformin has been reported to modulate cellular pathways involved in energy homeostasis, synaptic function, and inflammatory responses, including AMPK‐ and mTOR‐related signaling (Foretz et al. 2023; Sharma and Mehan 2021). These pathways are relevant to neurodevelopment and have also been implicated in experimental ASD‐related phenotypes. Consistent with this biological rationale, preclinical studies have reported that metformin can attenuate repetitive and social behavioral alterations and modulate oxidative and cholinergic parameters in ASD models (Elnahas et al. 2022). In clinical settings, metformin has also been investigated, particularly for the management of antipsychotic‐associated metabolic adverse effects in individuals with ASD (Anagnostou et al. 2016; Aman et al. 2018).

Despite these findings, important questions remain regarding the consistency of metformin responses across experimental models, sexes, and developmental windows. Previous preclinical investigations of metformin in prenatal VPA models have predominantly evaluated male rat offspring (Elnahas et al. 2022; Ishola et al. 2020; Atia et al. 2023), whereas metformin studies in mice have used other ASD‐related models, such as BTBR mice (Wang et al. 2018). Although sex‐related differences have been demonstrated in prenatal VPA models (Scheggi et al. 2020), comparatively little is known about the behavioral and biochemical responses to metformin in VPA‐exposed male and female mice evaluated within the same experimental design. In particular, previous metformin studies assessing oxidative stress and AChE activity in the prenatal VPA model were conducted in male rats (Ishola et al. 2020), leaving patterns across sexes and brain regions insufficiently characterized.

Given the involvement of the PFC and HPC in behavioral domains affected by prenatal VPA exposure, assessing redox‐related markers and AChE activity in these regions may help determine whether the behavioral effects of metformin are accompanied by biochemical changes. Because previous preclinical studies of metformin in ASD‐related models have largely focused on male animals, its effects in male and female offspring remain insufficiently characterized. We, therefore, hypothesized that metformin would attenuate VPA‐associated behavioral and biochemical alterations in both male and female offspring. We further explored whether treatment responses showed different patterns across sexes and, for biochemical outcomes, across brain regions. The objective of the present study was to evaluate the effects of metformin administered from PND21 to PND35 on locomotor/exploratory activity, repetitive behavior, anxiety‐like behavior, sociability, and spatial working memory in male and female mice prenatally exposed to VPA. In parallel, GSH, malondialdehyde (MDA), total protein content, and AChE activity were assessed in the PFC and HPC to characterize metformin‐associated biochemical patterns across sexes and brain regions.

## 2 Methods

### 2.1 Animals

Male and female Swiss mice (20–30 g, 8–12 weeks) were obtained from the Bioterium Center of the Institute of Biomedical Sciences, State University of Ceará. Animals were housed under controlled macroenvironmental conditions at 20–26°C, in accordance with recommendations of the Brazilian National Council for the Control of Animal Experimentation (CONCEA), under a 12‐h light/12‐h dark cycle, with food and water available ad libitum.

### 2.2 Ethical Statements

All procedures were approved by the Animal Ethics Committee (CEUA/UECE; NUP 31032.000519/2023‐49) and conducted in accordance with CONCEA guidelines, Brazilian regulations for animal research (National Council for the Control of Animal Experimentation (CONCEA) 2022), and ARRIVE 2.0 recommendations (Percie du Sert et al. 2020). No unexpected or significant hazards were associated with the procedures reported in this study. All experimental protocols complied with institutional and national ethical guidelines for animal research.

### 2.3 Drugs and Reagents

Metformin HCl (Fagron Micro, Brazil; purity 98.5%–101.0%) was prepared in distilled water (40 mg/mL). Depakene (Abbott; 500 mg VPA) was diluted in 0.1% Tween 80 (Sigma‐Aldrich) to 45 mg/mL. Other reagents were purchased from Sigma‐Aldrich (St. Louis, MO, USA): 50% trichloroacetic acid (TCA), DTNB, 35% perchloric acid, 1.2% TBA, acetylthiocholine iodide (ATC), Coomassie Brilliant Blue G‐250, and bovine serum albumin (BSA).

### 2.4 ASD Induction and Metformin Treatment

ASD was induced by intraperitoneal (i.p.) injection of VPA (450 mg/kg) in pregnant females on gestational day 12.5, using a 13 × 0.42 mm^2^ needle in the lower right abdominal quadrant (Al Sagheer et al. 2018). Control dams received 0.1% Tween 80 (i.p.).

Offspring were treated orally with metformin (400 mg/kg in 0.01 mL/g) from postnatal day 21 (PND21) to PND35. The metformin dose was selected on the basis of previous preclinical studies reporting behavioral and biochemical effects of metformin at comparable doses in rodent models relevant to ASD (Deng et al. 2022). The treatment was initiated at PND21, immediately after weaning, in accordance with previous studies using postnatal metformin intervention in prenatal VPA models (Ishola et al. 2020). This developmental window was selected to provide repeated pharmacological exposure during the juvenile/post‐weaning period, when behavioral maturation and synaptic refinement are still ongoing, while allowing behavioral assessment shortly after treatment. Control and VPA animals received an equivalent volume of distilled water by oral gavage following the same administration schedule.

### 2.5 Litter Allocation and Randomization

Prenatal exposure was assigned at the maternal level; therefore, all offspring from a given dam were exposed to the same prenatal condition (Control or VPA). After weaning, offspring were sexed. All pups born to Control dams remained in the Control condition and were separated according to sex. In contrast, offspring from VPA‐exposed dams were first separated by sex and then randomly allocated, by lot drawing, to either the VPA or VPA + Metformin postnatal condition. Randomization was performed within each litter and separately for males and females, with littermates distributed as equally as possible between the two groups. When an odd number of offspring of a given sex was available within a litter, allocation was kept as balanced as possible. Thus, littermates from the same VPA‐exposed litter could contribute to both the VPA and VPA + Metformin groups.

Four independent Control litters and six independent VPA‐exposed litters contributed offspring to the final experimental sample. Within the final sample, each Control litter contributed 3–9 offspring, whereas each VPA‐exposed litter contributed 1–6 offspring to the VPA group and 2–5 offspring to the VPA + Metformin group. Litter identity was retained throughout the experiment and included as a random effect in the generalized linear mixed models (GLMMs) to account for the non‐independence of littermates.

### 2.6 Experimental Design and Treatment Groups

Offspring were randomized into six groups:

$$\begin{array}{ccc} & & \mathrm{Control}\;\left(\mathrm{females}\;\mathrm{and}\;\mathrm{males}\right):\;\mathrm{prenatal}\;0.1\%\;\mathrm{Tween}\;80\;\left(i.p.\right)\\ & & +\;\mathrm{postnatal}\;\mathrm{distilled}\;\mathrm{water}\;\left(\mathrm{orally}\right). \end{array}$$

$$\begin{array}{ccc} & & \mathrm{VPA}\;\left(\mathrm{females}\;\mathrm{and}\;\mathrm{males}\right):\;\mathrm{prenatal}\;\mathrm{VPA}\;\left(450\;\mathrm{mg}/\mathrm{kg},\;i.p.\right)\\ & & +\;\mathrm{postnatal}\;\mathrm{distilled}\;\mathrm{water}\;\left(\mathrm{orally}\right). \end{array}$$

$$\begin{array}{ccc} & & \mathrm{VPA}+\mathrm{Metformin}\;\left(\mathrm{females}\;\mathrm{and}\;\mathrm{males}\right):\;\mathrm{prenatal}\;\mathrm{VPA}\;\\ & & \left(450\;\mathrm{mg}/\mathrm{kg},\;i.p.\right)\\ & & +\mathrm{postnatal}\;\mathrm{metformin}\;\left(400\;\mathrm{mg}/\mathrm{kg},\;\mathrm{orally}\right). \end{array}$$

The final distribution was as follows: Control females, *n* = 10 from 4 litters; Control males, *n* = 14 from 4 litters; VPA females, *n* = 10 from 6 litters; VPA males, *n* = 9 from 5 litters; VPA + Metformin females, *n* = 11 from 6 litters; and VPA + Metformin males, *n* = 9 from 6 litters. The same VPA‐exposed litters contributed offspring to both postnatal VPA conditions whenever animals of the corresponding sex were available. The experimental design was focused on evaluating metformin as an intervention in VPA‐exposed offspring; therefore, a separate Control + Metformin group was not included.

### 2.7 Body Weight, Litter Outcomes, and Survival Monitoring

Body weight was recorded daily throughout the 15‐day postnatal treatment period (PND21–PND35) in both male and female offspring. Daily group‐level mean body weights were retained in the experimental records and were, therefore, used for descriptive evaluation of body‐weight trajectories over the treatment period.

Litter‐related outcomes and survival were also monitored throughout the experiment. For litters that survived and remained available for post‐weaning follow‐up, the number of offspring per litter was recorded. Maternal deaths following prenatal treatment, complete litter losses, and offspring deaths occurring during the postnatal treatment period were documented. In cases of complete litter loss due to cannibalism, the event was identified during routine cage inspection after the litter had already been consumed or only pup remains were present. Consequently, the original number of offspring in these litters could not be reliably determined, and estimates of offspring number per litter were restricted to litters that survived and could be followed until the post‐weaning period.

Offspring survival during the treatment period was calculated for each experimental group as the proportion of animals remaining alive at the end of treatment relative to the number of animals allocated to that group at treatment initiation.

### 2.8 Behavioral Tests

Behavioral tests were conducted during the last 2 days of treatment. On PND34 (the 14th day of treatment), the open field, self‐grooming, and elevated plus maze (EPM) tests were performed. On PND35 (the 15th day of treatment), the three‐chamber social interaction and Y‐maze tests were conducted. Behavioral tests were conducted in a fixed sequence from less to more stressful paradigms, with adequate intervals between tests to minimize carryover effects. All apparatuses were cleaned with 5% alcohol between sessions to minimize olfactory interference. All behavioral assessments were performed by experimenters blinded to the treatment groups in order to reduce observational bias.

### 2.9 General Exploratory Activity—Open Field Test

The open field test was performed in a transparent acrylic arena divided into nine equal quadrants. Mice were observed for 5 min following a 1‐min habituation. The number of crossings (all four paws entering a new quadrant) and rearings (animals standing on hind limbs) were recorded as measures of locomotor and exploratory activity, respectively. Higher numbers of crossings were interpreted as increased locomotor activity, whereas rearings were considered an index of vertical exploratory behavior (Archer 1973).

### 2.10 Stereotypic and Repetitive Behavior—Self‐Grooming Test

Self‐grooming behavior was assessed in the open field apparatus after a 1‐min habituation period. Mice were observed for 10 min, and the total number of grooming events and the total duration of grooming behavior (s) were recorded. Higher numbers of grooming events and/or longer grooming duration were interpreted as increased repetitive self‐grooming behavior (Silverman et al. 2010).

### 2.11 Anxiety‐Like Behavior—EPM Test

The EPM consists of a cross‐shaped acrylic maze elevated 50 cm above the floor, with two open and two closed arms (30 × 5 cm^2^; closed walls: 15 cm high). Mice were placed at the center facing an open arm and allowed to explore for 5 min. The number of entries and time spent in each type of arm were recorded. Greater open‐arm entries and time spent in the open arms indicate reduced anxiety‐like behavior, whereas preference for closed arms reflects higher anxiety levels (Lister 1987). An anxiety index was also calculated to integrate open‐arm entries and time spent in the open arms, according to the following formula: *anxiety index = 1 − {[time spent in open arms/total test duration] +* *[number of open‐arm entries/total arm entries]}/2*. Higher anxiety index values indicate greater anxiety‐like behavior, whereas lower values indicate reduced anxiety‐like behavior (Cohen et al. 2013).

### 2.12 Social Behavior—Three‐Chamber Social Interaction Test

The apparatus is an acrylic box (60 × 40 × 22 cm^3^) divided into three interconnected chambers. Each lateral chamber contains a cylindrical acrylic cage: one with an unfamiliar conspecific (social stimulus) and the other empty (nonsocial stimulus). After habituation in the central chamber, the animal explored freely for 5 min. Time spent and number of entries into each chamber were recorded. Greater time in the social chamber indicates higher sociability, whereas preference for the nonsocial chamber suggests social deficits (Yang et al. 2011). Sociability was assessed by comparing the time spent in the social and nonsocial chambers, whereas time spent in the central chamber was not considered a measure of social preference. A social preference index (SPI) was additionally calculated as follows: *SPI = (Tsocial − Tnonsocial)/(Tsocial + Tnonsocial)*, where Tsocial and Tnonsocial represent the time spent in the social and nonsocial chambers, respectively. Positive SPI values indicate preference for the social stimulus, values around zero indicate no preference, and negative values indicate preference for the nonsocial stimulus (Rein et al. 2020).

### 2.13 Working Memory—Y‐Maze Test

The Y‐maze consists of three acrylic arms (75.5 × 11.7 × 34.5 cm^3^) arranged at 120° angles. Mice were placed at the end of one arm and allowed to explore freely for 8 min. The sequence and total number of arm entries were recorded. Spontaneous alternation (consecutive entries into all three arms without repetition) was calculated as:

%alternation=(actualalternations/[totalentries−2])×100.

Higher percentages indicate better short‐term spatial working memory, whereas lower values suggest cognitive impairment (Prieur and Jadavji 2019).

### 2.14 Euthanasia and Brain Area Dissection

Animals were euthanized by rapid decapitation, followed by immediate brain removal to minimize postmortem changes in the biochemical parameters analyzed (Ko et al. 2019). The PFC and HPC were dissected on ice, homogenized in PBS, and processed for total protein content, oxidative stress markers, and acetylcholinesterase activity. Biochemical analyses were performed under blinded conditions; samples were identified only by color‐coded labels, and the experimenters conducting the assays were unaware of the corresponding experimental groups until data collection was completed. Equipment was cleaned and sanitized between procedures to prevent cross‐contamination.

### 2.15 Total Protein

Total protein content was measured by the Bradford method (Bradford 1976), based on the binding of Coomassie Brilliant Blue G‐250 to basic and aromatic amino acid residues. A standard curve was prepared using serial dilutions of bovine serum albumin (BSA). Bradford reagent (diluted 1:5 in distilled water) was added (200 µL) to each well containing 40 µL of 1:10 diluted sample or standard. After 5 min of incubation, absorbance was read at 595 nm.

### 2.16 Oxidative Stress Assessment—Lipid Peroxidation (MDA) and Antioxidant Defense (GSH)

Lipid peroxidation was quantified by the TBARS method. Tissue homogenates in 150 mM phosphate buffer (63 µL) were mixed with 35% perchloric acid (100 µL), centrifuged (5000 rpm, 10 min, and 4°C), and the supernatant (150 µL) incubated with 1.2% TBA (50 µL) at 95°C for 30 min. After cooling, absorbance was measured at 535 nm in a 96‐well plate (Nagababu et al. 2010).

GSH levels were determined by mixing 10% homogenate (65.57 µL) with distilled water (52.45 µL) and 50% TCA (13.11 µL), followed by centrifugation (5000 rpm, 15 min, and 4°C). Supernatant (65.57 µL) was added to Tris‐HCl buffer (131.14 µL) in a 96‐well plate, followed by DTNB (3.27 µL), and absorbance was read at 412 nm within 5 min (Sedlak and Lindsay 1968).

### 2.17 Acetylcholinesterase (AChE) Activity

AChE activity was determined according to the method of Ellman et al. (1961), based on the hydrolysis of acetylthiocholine (ATC) into thiocholine, which reacts with DTNB to form a yellow chromophore. In 96‐well plates, 34 µL of tissue homogenate and 134 µL of DTNB (0.75 mM) were added to each well. Absorbance was initially recorded at 412 nm before the addition of ATC and used as the blank reading. Subsequently, 34 µL of ATC (9 mM) was added, and absorbance was recorded at 412 nm from 1 to 5 min. Absorbance values at 1 and 5 min were corrected by subtracting the respective blank absorbance, and the rate of change in absorbance was calculated as Δ*A*/mi *n* = (*A*_5_ corrected − *A*_1_ corrected)/4. The concentration of ATC hydrolyzed was calculated according to the Beer–Lambert equation using an extinction coefficient of 13.6 mM^−1^ cm^−1^ and an optical path length of 0.642 cm, as follows: *c* = (Δ*A*/min × 1000)/(13.6 × 0.642). Specific AChE activity was subsequently calculated as *c*/[(*VA*/*VT*) × *p*], where *VA* represents the sample volume (34 µL), *VT* the final reaction volume (200 µL), and *p* the protein concentration of the sample (mg/mL). AChE activity was expressed as nmol of ATC hydrolyzed per minute per milligram of protein (nmol/min/mg protein) (Isomae et al. 2002).

### 2.18 Statistical Analysis

Statistical analyses for behavioral parameters were performed using GLMMs with a Gamma distribution, implemented in R software (R Foundation for Statistical Computing, Vienna, Austria). Experimental group (Control, VPA, and VPA + Metformin), sex (female and male), and the group × sex interaction were included as fixed effects. Litter was included as a random effect to account for intra‐litter dependency. Post hoc pairwise comparisons were performed on the basis of estimated marginal means using the emmeans package, with Tukey adjustment for multiple comparisons. Comparisons between experimental groups within each sex were conducted as prespecified simple‐effect analyses. Comparisons between males and females were also performed within each experimental group. Differences in treatment response between males and females were interpreted as sex‐dependent only when supported by a significant group × sex interaction. Effect estimates were expressed as mean ratios (MRs) with corresponding 95% confidence intervals (95% CIs) and Tukey‐adjusted *p* values. Statistical significance was set at *p* < 0.05.

The derived behavioral indices were analyzed separately from the individual behavioral parameters. The anxiety index and SPI were analyzed using linear mixed‐effects models (LMMs) in R. Experimental group (Control, VPA, and VPA + Metformin), sex, and the group × sex interaction were included as fixed effects, whereas litter was included as a random intercept. When indicated by model diagnostics, heterogeneity of residual variance among experimental groups was accommodated using a heterogeneous variance structure (varIdent) implemented with the nlme package. Fixed effects were evaluated using analysis of variance, and post hoc pairwise comparisons were performed on the basis of estimated marginal means using the emmeans package with Tukey adjustment for multiple comparisons. Comparisons among experimental groups were performed within each sex, and comparisons between males and females were performed within each experimental group. For pairwise comparisons derived from these LMMs, effect estimates were expressed as estimated mean differences (MDs), together with Tukey‐adjusted 95% confidence intervals (95% CIs) and *p* values. Statistical significance was set at *p* < 0.05.

For biochemical parameters, statistical analyses were performed using GraphPad Prism version 9.5.0 (GraphPad Software, San Diego, CA, USA). Biochemical comparisons were performed separately within each sex and brain region. Data normality was assessed using the Shapiro–Wilk test. For normally distributed variables, one‐way ANOVA followed by Tukey's post hoc test was applied. Non‐parametric data were analyzed using the Kruskal–Wallis test followed by Dunn's post hoc test. Results are expressed as mean ± standard error of the mean (SEM), and statistical significance was set at *p* < 0.05.

## 3 Results

### 3.1 Maternal and Litter Outcomes

Fifteen pregnant dams were exposed to VPA. Three dams (20.0%) died following VPA administration. Among the 12 surviving VPA‐exposed dams, 6 (50.0%) subsequently experienced complete litter loss due to cannibalism. No maternal deaths or complete litter losses were recorded in the Control condition. Consequently, four Control litters and six VPA‐exposed litters were available for post‐weaning follow‐up. Because complete litter losses due to cannibalism were identified only after the litters had already been consumed, the original number of pups in these litters could not be reliably determined. Therefore, offspring number per litter in the VPA condition was restricted to the six surviving litters available for post‐weaning follow‐up (Table 1).

**TABLE 1:** Maternal and litter outcomes following prenatal exposure.

Outcome	Control	Prenatal VPA cohort
Pregnant dams initially included, *n*	4	15
Maternal deaths following prenatal exposure, *n*/*N* (%)	0/4 (0%)	3/15 (20.0%)
Complete litter loss due to cannibalism, *n*/*N* (%)	0/4 (0%)	6/12 surviving dams (50.0%)
Litters available for post‐weaning follow‐up, *n*	4	6
Offspring per surviving litter at treatment onset, mean ± SD (range)	6.0 ± 1.63 (4–8)	7.5 ± 2.88 (5–13)
Female offspring per surviving litter at treatment onset, mean ± SD (range)	2.5 ± 1.29 (1–4)	4.17 ± 1.47 (3–7)
Male offspring per surviving litter at treatment onset, mean ± SD (range)	3.5 ± 1.73 (2–5)	3.33 ± 1.63 (1–6)

*Note*: Maternal deaths refer to deaths occurring shortly after prenatal exposure, whereas dams were still pregnant and before delivery; for this outcome, *n* represents the number of maternal deaths and *N* the number of pregnant dams initially included. Complete litter loss refers to postnatal loss due to cannibalism; for this outcome, *n* represents the number of completely lost litters and *N* the number of dams that survived prenatal exposure and gave birth. Litters available for post‐weaning follow‐up correspond to litters with surviving offspring. Offspring‐per‐litter and sex‐specific offspring counts were calculated from these surviving litters at treatment onset and are presented as mean ± SD (range). Data are presented as *n*/*N* (%), number of litters (*n*), or mean ± SD (range), as appropriate.

Abbreviation: VPA, valproic acid.

Among litters available for post‐weaning follow‐up, the mean number of offspring per litter was 6.0 ± 1.63 in the Control condition and 7.5 ± 2.88 in the prenatal VPA‐exposed condition.

### 3.2 Postnatal Survival and Final Analyzed Sample

Following weaning and sex‐specific randomization of VPA‐exposed offspring into the VPA and VPA + Metformin groups, 69 offspring entered the 15‐day postnatal treatment period: 24 in the Control group, 23 in the VPA group, and 22 in the VPA + Metformin group. No deaths occurred among Control offspring during treatment. In the VPA group, four animals died, comprising three females and one male, whereas two deaths occurred in the VPA + Metformin group, comprising one female and one male. Sex‐specific survival rates and the final number of animals and contributing litters are presented in Table 2.

**TABLE 2:** Postnatal survival during the 15‐day treatment period and final analyzed sample, stratified by sex.

Sex	Parameter	Control	VPA	VPA + Metformin
**Female**	Animals at treatment onset, *n*	10	13	12
	Deaths during treatment, *n*	0	3	1
	Survival during treatment, %	100.0	76.9	91.7
	Animals included in final analysis, *n*	10	10	11
	Contributing litters in final analysis, *n*	4	6	6
**Male**	Animals at treatment onset, *n*	14	10	10
	Deaths during treatment, *n*	0	1	1
	Survival during treatment, %	100.0	90.0	90.0
	Animals included in final analysis, *n*	14	9	9
	Contributing litters in final analysis, *n*	4	5	6

*Note*: Offspring were allocated to the Control, VPA, or VPA + Metformin groups after weaning, as described in Section 2. Survival was calculated as the number of animals remaining alive at the end of the treatment period relative to the number allocated to each group at treatment onset. The final analyzed sample corresponds to the animals that survived the treatment period and were included in the subsequent analyses. The number of contributing litters is reported separately for females and males.

Abbreviation: VPA, valproic acid.

Overall survival during the treatment period was 100% in the Control group (24/24), 82.6% in the VPA group (19/23), and 90.9% in the VPA + Metformin group (20/22). The final analyzed sample therefore comprised 63 offspring: 24 Controls, 19 VPA‐exposed offspring receiving vehicle, and 20 VPA‐exposed offspring treated with metformin. The final sample included 31 females and 32 males, with multiple independent litters contributing to each sex‐specific experimental group.

### 3.3 Body Weight During Postnatal Treatment

Body weight was monitored throughout the 15‐day postnatal treatment period. Mean body weight increased from D1 to D15 in all experimental groups and in both sexes. Initial and final mean body weights, as well as the descriptive change over the treatment period, are presented in Table 3.

**TABLE 3:** Body weight during the 15‐day postnatal treatment period in female and male offspring.

Sex	Body‐weight parameter	Control	VPA	VPA + Metformin
**Female**	D1 mean body weight (g)	12.50	10.20	10.77
	D15 mean body weight (g)	19.05	16.83	15.27
	Change D15–D1 (g)	+6.55	+6.63	+4.50
**Male**	D1 mean body weight (g)	14.60	11.75	11.75
	D15 mean body weight (g)	24.15	19.95	17.00
	Change D15–D1 (g)	+9.55	+8.20	+5.25

*Note*: Body weight is expressed in grams (g) and presented as group‐level means for D1 and D15 in the Control, VPA, and VPA + Metformin groups. The change in mean body weight was calculated as D15–D1. Only group‐level mean values were retained in the experimental records; therefore, individual‐level measures of variability (SD or SEM) and inferential statistical comparisons could not be performed. Because deaths occurred during treatment in the VPA‐exposed groups, the number of animals contributing to the group means may have differed between D1 and D15.

Abbreviation: VPA, valproic acid.

In females, mean body weight increased from 12.50 to 19.05 g in the Control group, from 10.20 to 16.83 g in the VPA group, and from 10.77 to 15.27 g in the VPA + Metformin group. In males, mean body weight increased from 14.60 to 24.15 g in the Control group, from 11.75 to 19.95 g in the VPA group, and from 11.75 to 17.00 g in the VPA + Metformin group. The corresponding descriptive increases in mean body weight were 6.55, 6.63, and 4.50 g in females and 9.55, 8.20, and 5.25 g in males, respectively. Because only group‐level mean values were retained, body‐weight data were interpreted descriptively, and no inferential statistical comparisons were performed.

### 3.4 Behavioral Tests

Behavioral outcomes were assessed using the open field, self‐grooming, EPM, three‐chamber social interaction, and Y‐maze tests. Experimental group, sex, and the group × sex interaction were evaluated as described in Section 2.18. Descriptive values for each behavioral parameter according to experimental group and sex are presented in Table 4.

**TABLE 4:** Descriptive analysis of behavioral parameters evaluated in female and male mice prenatally exposed to valproic acid (VPA) and treated with metformin.

		Females			Males		
Test	Parameter	Control	VPA	VPA + Metformin	Control	VPA	VPA + Metformin
**Open field**	Number of crossings	127.5 ± 15.0 (*n* = 10)	82.7 ± 28.2 (*n* = 10)	122.1 ± 25.8 (*n* = 11)	127.5 ± 33.9 (*n* = 14)	82.7 ± 29.1 (*n* = 9)	122.1 ± 26.0 (*n* = 9)
	Number of rearing events	5.3 ± 2.5 (*n* = 10)	17.5 ± 6.8 (*n* = 10)	4.3 ± 1.3 (*n* = 11)	4.3 ± 2.8 (*n* = 14)	9.1 ± 2.6 (*n* = 9)	1.7 ± 1.3 (*n* = 9)
**Grooming**	Number of grooming events	6.0 ± 2.1 (*n* = 10)	14.6 ± 7.0 (*n* = 10)	6.7 ± 2.2 (*n* = 11)	6.3 ± 2.6 (*n* = 14)	15.4 ± 2.9 (*n* = 9)	8.8 ± 2.0 (*n* = 9)
	Grooming duration (s)	79.0 ± 48.2 (*n* = 10)	100.8 ± 31.8 (*n* = 10)	48.6 ± 23.1 (*n* = 11)	48.0 ± 20.5 (*n* = 14)	140.3 ± 19.7 (*n* = 9)	72.3 ± 22.5 (*n* = 9)
**Elevated plus maze**	Number of entries into open arms	15.4 ± 4.1 (*n* = 10)	11.7 ± 2.4 (*n* = 10)	19.3 ± 2.6 (*n* = 11)	18.6 ± 3.2 (*n* = 14)	13.9 ± 3.7 (*n* = 9)	17.4 ± 4.4 (*n* = 9)
	Number of entries into closed arms	13.3 ± 2.5 (*n* = 10)	16.0 ± 2.8 (*n* = 10)	12.6 ± 2.9 (*n* = 11)	12.1 ± 1.7 (*n* = 14)	15.8 ± 3.2 (*n* = 9)	10.7 ± 3.2 (*n* = 9)
	Time spent in open arms (s)	143.3 ± 26.0 (*n* = 10)	124.7 ± 33.8 (*n* = 10)	147.9 ± 19.7 (*n* = 11)	152.4 ± 21.0 (*n* = 14)	119.1 ± 20.0 (*n* = 9)	154.1 ± 33.1 (*n* = 9)
	Time spent in closed arms (s)	109.2 ± 25.2 (*n* = 10)	122.4 ± 24.2 (*n* = 10)	102.6 ± 32.9 (*n* = 11)	98.9 ± 19.2 (*n* = 14)	130.7 ± 6.7 (*n* = 9)	110.8 ± 28.7 (*n* = 9)
**Social interaction**	Number of entries into the social compartment	13.5 ± 3.2 (*n* = 10)	7.2 ± 2.3 (*n* = 10)	12.4 ± 2.5 (*n* = 11)	14.1 ± 2.0 (*n* = 14)	8.1 ± 1.3 (*n* = 9)	11.3 ± 0.9 (*n* = 9)
	Number of entries into the nonsocial compartment	8.3 ± 2.0 (*n* = 10)	8.8 ± 1.4 (*n* = 10)	7.1 ± 3.0 (*n* = 11)	7.6 ± 1.7 (*n* = 14)	11.2 ± 1.6 (*n* = 9)	7.2 ± 1.9 (*n* = 9)
	Time spent in the social compartment (s)	145.4 ± 19.6 (*n* = 10)	114.1 ± 18.0 (*n* = 10)	136.7 ± 25.7 (*n* = 11)	159.8 ± 22.4 (*n* = 14)	107.1 ± 10.8 (*n* = 9)	114.9 ± 19.0 (*n* = 9)
	Time spent in the nonsocial compartment (s)	78.9 ± 13.7 (*n* = 10)	99.2 ± 22.7 (*n* = 10)	77.3 ± 16.9 (*n* = 11)	71.5 ± 12.0 (*n* = 14)	109.1 ± 15.0 (*n* = 9)	97.1 ± 19.8 (*n* = 9)
**Y‐maze**	Correct triads (%)	88.6 ± 6.7 (*n* = 10)	45.5 ± 8.9 (*n* = 10)	67.4 ± 13.9 (*n* = 11)	89.7 ± 5.1 (*n* = 14)	39.0 ± 4.3 (*n* = 9)	67.9 ± 7.6 (*n* = 9)

*Note*: Data are presented as mean ± standard deviation (SD), with the number of animals indicated in parentheses (*n*), stratified by sex and experimental group. In the social interaction test, “social compartment” refers to the compartment containing the unfamiliar conspecific (social stimulus), whereas “nonsocial compartment” refers to the compartment without the social stimulus.

### 3.5 Locomotor and Vertical Exploratory Activity—Open Field Test

**FIGURE 1:**
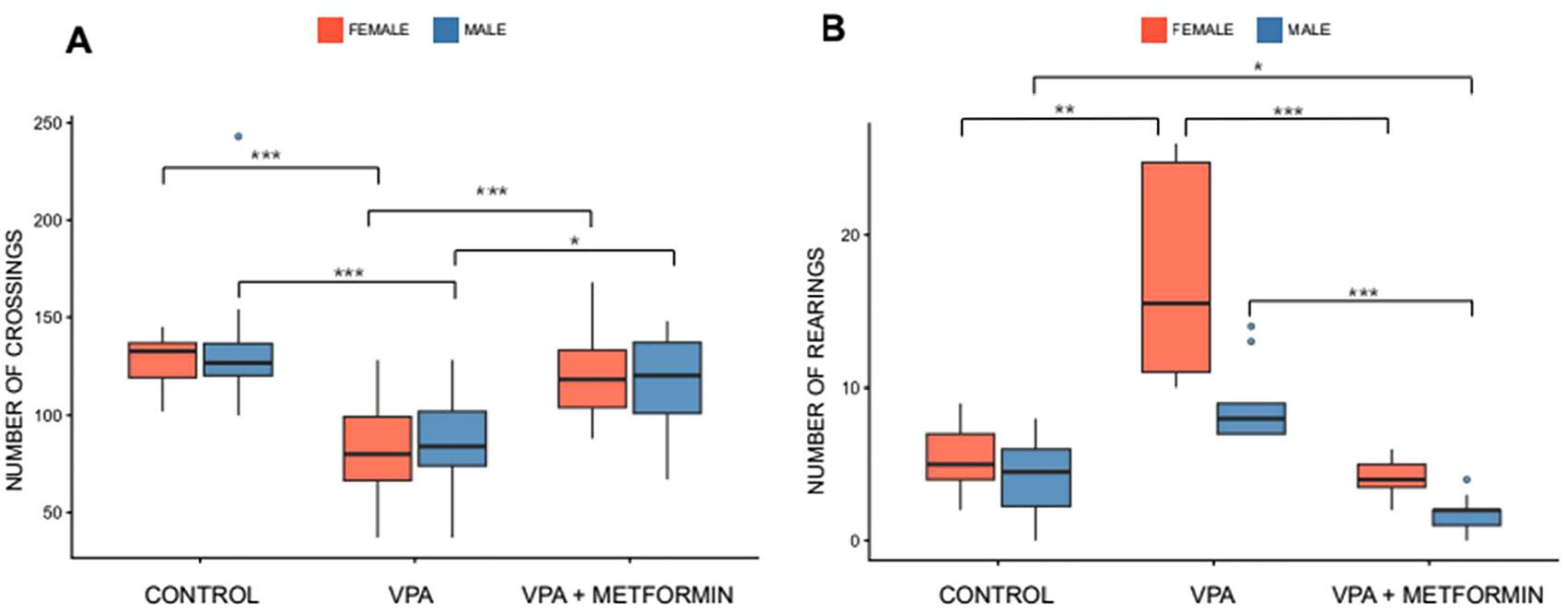
Open field test performance in female and male mice prenatally exposed to VPA and treated with metformin: (A) number of crossings and (B) number of rearing events in the Control, VPA, and VPA + Metformin groups. Group sizes were females—Control (*n* = 10), VPA (*n* = 10), and VPA + Metformin (*n* = 11); males—Control (*n* = 14), VPA (*n* = 9), and VPA + Metformin (*n* = 9). Data are shown as boxplots, with the central line representing the median, boxes indicating the interquartile range, whiskers representing data dispersion, and dots indicating outliers. Statistical analyses were performed using generalized linear mixed models (GLMMs) with a Gamma distribution, with litter included as a random effect. VPA, valproic acid. Significant differences are indicated as *p* < 0.05 (*), *p* < 0.01 (**), and *p* < 0.001 (***).

Locomotor activity, assessed by the number of *crossings* in the open field test, was reduced in animals exposed to VPA compared with the Control group (Figure 1A). In the overall analysis, the VPA group showed approximately 36% fewer *crossings* than the Control group, as indicated by the MR VPA/Control = 0.64, 95% CI [0.49–0.85]; *p* = 0.002.

Treatment with metformin increased locomotor activity compared with the VPA group. The MR VPA + Metformin/VPA was 1.54, 95% CI [1.26–1.89]; *p* < 0.001, indicating that treated animals exhibited approximately 54% more *crossings* than untreated VPA animals. No difference was observed between the VPA + Metformin and Control groups, with an MR VPA + Metformin/Control = 0.99, 95% CI [0.75–1.30]; *p* = 0.923.

In sex‐specific simple‐effect analyses, a similar pattern was observed in females and males, and descriptive values (mean ± SD) for each experimental group are presented in Table 4. In females, the VPA group showed a lower number of *crossings* compared with the Control group, with an MR VPA/Control = 0.65, 95% CI [0.49–0.85]; *p* < 0.001, and also compared with the VPA + Metformin group, with an MR VPA/VPA + Metformin = 0.67, 95% CI [0.52–0.86]; *p* < 0.001. No difference was observed between the Control and VPA + Metformin groups, with an MR Control/VPA + Metformin = 1.03, 95% CI [0.79–1.35]; *p* = 0.959.

In males, the VPA group also showed reduced locomotor activity compared with the Control group, with an MR VPA/Control = 0.63, 95% CI [0.48–0.82]; *p* < 0.001. Compared with the VPA + Metformin group, the MR VPA/VPA + Metformin was 0.72, 95% CI [0.55–0.94]; *p* = 0.012, indicating lower activity in VPA animals. No significant difference was observed between the Control and VPA + Metformin groups, with an MR Control/VPA + Metformin = 1.15, 95% CI [0.88–1.50]; *p* = 0.434.

Nosignificantgroup×sexinteractiontermswereobservedforthenumberofcrossings(p>0.05).

Vertical exploratory activity, assessed by the number of *rearing* events in the open field test, was altered in VPA‐exposed animals compared with the Control group (Figure 1B). In the overall analysis, the VPA group showed a higher number of *rearing* events than the Control group, with an MR VPA/Control = 3.30, 95% CI [1.44–7.57]; *p* = 0.005.

Treatment with metformin reduced *rearing* behavior compared with the VPA group. The MR VPA + Metformin/VPA was 0.24; *p* < 0.001, indicating a marked reduction in vertical exploratory activity in treated animals. No difference was observed between the VPA + Metformin and Control groups, with an MR VPA + Metformin/Control = 0.81, 95% CI [0.36–1.81]; *p* = 0.602.

In sex‐specific simple‐effect analyses, descriptive values are presented in Table 4. In females, the VPA group showed a higher number of *rearing* events compared with the Control group, with an MR VPA/Control = 0.30, 95% CI [0.13–0.72]; *p* = 0.003, and compared with the VPA + Metformin group, with an MR VPA/VPA + Metformin = 0.24, 95% CI [0.11–0.57]; *p* < 0.001. No difference was observed between Control and VPA + Metformin groups, with an MR Control/VPA + Metformin = 1.24, 95% CI [0.53–2.87]; *p* = 0.82.

In males, no significant difference was observed between the VPA and Control groups (VPA/Control = 0.47, 95% CI [0.21–1.07]; *p* = 0.081). However, the VPA group showed higher *rearing* compared with the VPA + Metformin group, with an MR VPA/VPA + Metformin = 0.18, 95% CI [0.07–0.46]; *p* < 0.001. Additionally, a difference was observed between Control and VPA + Metformin groups, with an MR Control/VPA + Metformin = 2.56, 95% CI [1.13–5.83]; *p* = 0.02.

No significant group × sex interaction terms were observed for *rearing* behavior (*p* > 0.05).

### 3.6 Evaluation of Repetitive Behavior—Self‐Grooming Test

**FIGURE 2:**
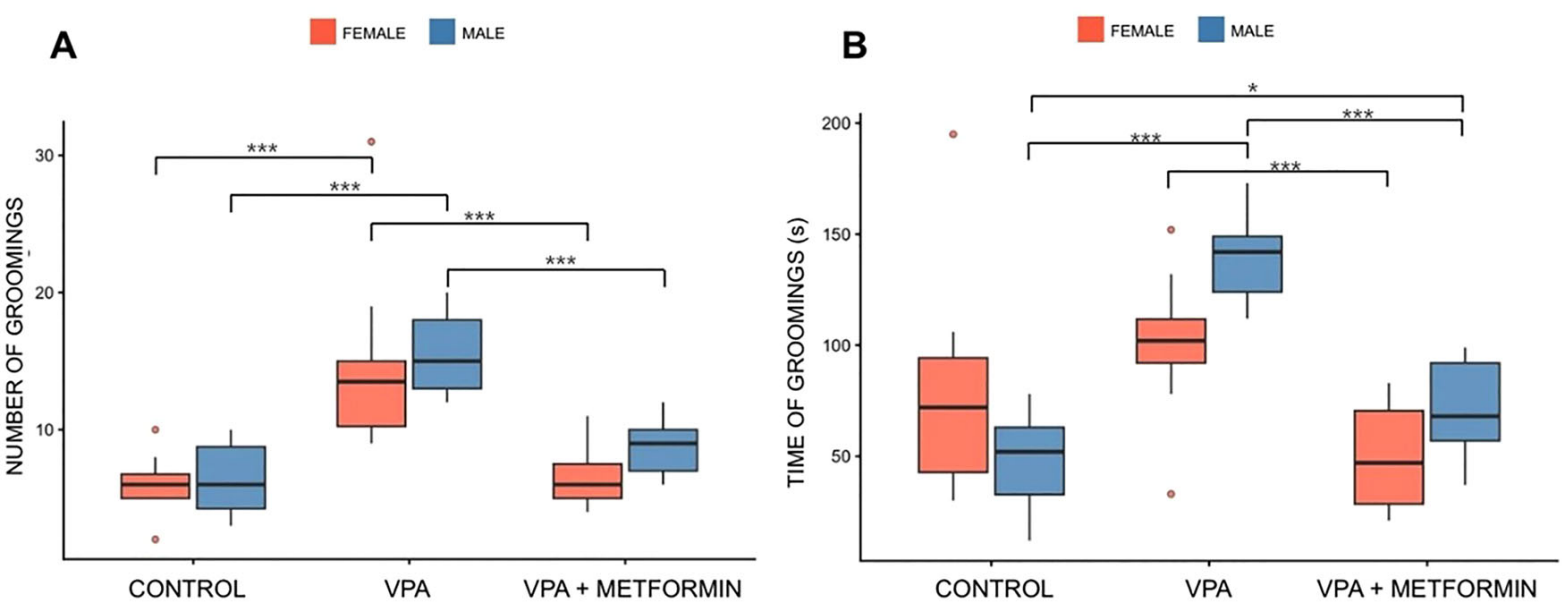
Grooming behavior in female and male mice prenatally exposed to VPA and treated with metformin: (A) number of grooming events and (B) time of grooming (s) in the Control, VPA, and VPA + Metformin groups. Group sizes were females—Control (*n* = 10), VPA (*n* = 10), and VPA + Metformin (*n* = 11); males—Control (*n* = 14), VPA (*n* = 9), and VPA + Metformin (*n* = 9). Data are shown as boxplots, with the central line representing the median, boxes indicating the interquartile range, whiskers representing data dispersion, and dots indicating outliers. Statistical analyses were performed using generalized linear mixed models (GLMMs) with a Gamma distribution, with litter included as a random effect. VPA, valproic acid.

Repetitive behavior, assessed by the number of grooming events in the open field test, was increased in animals exposed to VPA compared with the Control group (Figure 2A). In the overall analysis, the VPA group showed a higher number of grooming events than the Control group, as indicated by the MR VPA/Control = 2.39, 95% CI [1.72–3.31]; *p* < 0.001.

Treatment with metformin reduced grooming behavior compared with the VPA group. The MR VPA + Metformin/VPA was 0.46, 95% CI [0.35–0.61]; *p* < 0.001, indicating a marked decrease in repetitive behavior in treated animals. No difference was observed between the VPA + Metformin and Control groups, with an MR VPA + Metformin/Control = 1.11, 95% CI [0.81–1.53]; *p* = 0.523.

In sex‐specific simple‐effect analyses, a similar pattern was observed in females and males, and descriptive values (mean ± SD) for each experimental group are presented in Table 4. In females, the VPA group showed a higher number of grooming events compared with the Control group (VPA/Control = 2.44, 95% CI [1.72–3.45]; *p* < 0.001) and compared with the VPA + Metformin group (VPA/VPA + Metformin = 2.17; 95% CI [1.54–3.03]; *p* < 0.001). No difference was observed between the Control and VPA + Metformin groups (Control/VPA + Metformin = 0.89, 95% CI [0.64–1.25]; *p* = 0.712).

In males, the VPA group also showed increased grooming compared with the Control group (VPA/Control = 2.45, 95% CI [1.75–3.45]; *p* < 0.001) and compared with the VPA + Metformin group (VPA/VPA + Metformin = 1.76, 95% CI [1.22–2.50]; *p* < 0.001). A difference was observed between the Control and VPA + Metformin groups (Control/VPA + Metformin = 0.72, 95% CI [0.51–1.00]; *p* = 0.049), indicating that the VPA + Metformin group remained significantly different from the Control group. No significant effect of sex was observed on the number of grooming events (male/female = 1.05, 95% CI [0.80–1.37]; *p* = 0.727).

Grooming duration, assessed as time spent grooming, did not differ significantly between the VPA and Control groups in the overall analysis (VPA/Control = 1.70, 95% CI [0.99–2.94]; *p* = 0.056; Figure 2B).

Treatment with metformin reduced grooming duration compared with the VPA group, with an MR VPA + Metformin/VPA = 0.45, 95% CI [0.33–0.62]; *p* < 0.001. No difference was observed between the VPA + Metformin and Control groups (VPA + Metformin/Control = 0.77, 95% CI [0.45–1.31]; *p* = 0.329).

In sex‐specific simple‐effect analyses, descriptive values are presented in Table 4. In females, no difference was observed between the VPA and Control groups (VPA/Control = 1.54, 95% CI [0.90–2.63]; *p* = 0.143). However, the VPA group showed increased grooming time compared with the VPA + Metformin group (VPA/VPA + Metformin = 2.17, 95% CI [1.47–3.23]; *p* < 0.001). No difference was observed between Control and VPA + Metformin groups (Control/VPA + Metformin = 1.43, 95% CI [0.85–2.39]; *p* = 0.241).

In males, the VPA group showed increased grooming time compared with the Control group (VPA/Control = 3.23, 95% CI [1.92–5.26]; *p* < 0.001) and compared with the VPA + Metformin group (VPA/VPA + Metformin = 1.96, 95% CI [1.30–2.94]; *p* < 0.001). No difference was observed between the Control and VPA + Metformin groups (Control/VPA + Metformin = 0.62, 95% CI [0.37–1.03]; *p* = 0.069).

A significant overall effect of sex was observed for grooming duration (male/female = 1.39, 95% CI [1.01–1.91]; *p* = 0.042). In sex‐specific comparisons within experimental groups, Control males showed lower grooming duration than Control females (male/female = 0.49, 95% CI [0.32–0.77]; *p* = 0.002), whereas no difference was observed between males and females within the VPA + Metformin group (male/female = 1.12, 95% CI [0.71–1.76]; *p* = 0.621).

### 3.7 Evaluation of Anxiety‐Like Behavior—EPM

**FIGURE 3:**
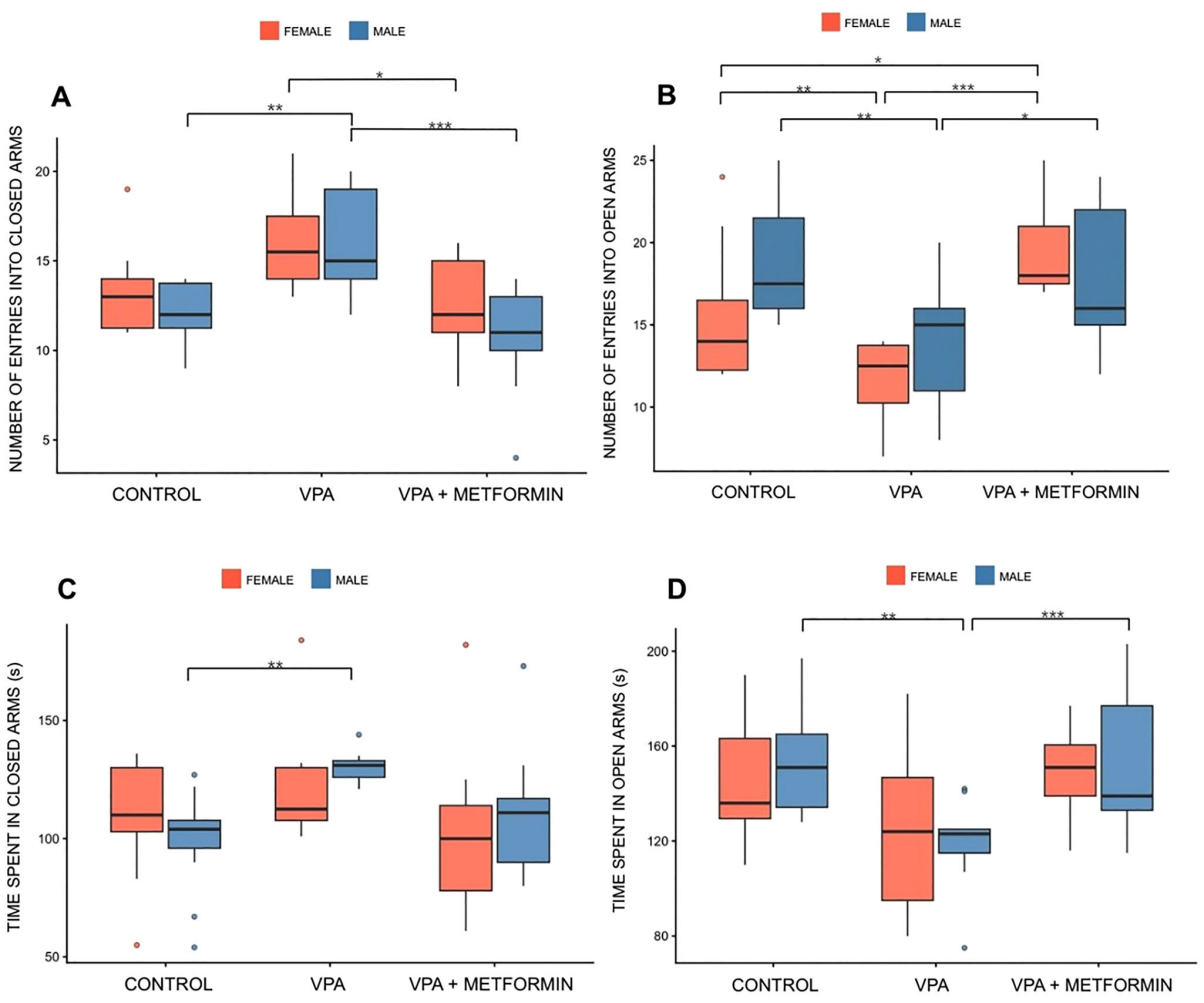
Elevated plus maze performance in female and male mice prenatally exposed to VPA and treated with metformin: (A) number of entries into closed arms, (B) number of entries into open arms, (C) time spent in closed arms, and (D) time spent in open arms in the Control, VPA, and VPA + Metformin groups. Group sizes were females—Control (*n* = 10), VPA (*n* = 10), and VPA + Metformin (*n* = 11); males—Control (*n* = 14), VPA (*n* = 9), and VPA + Metformin (*n* = 9). Data are shown as boxplots, with the central line representing the median, boxes indicating the interquartile range, whiskers representing data dispersion, and dots indicating outliers. Statistical analyses were performed using generalized linear mixed models (GLMMs) with a gamma distribution, with litter included as a random effect. VPA, valproic acid. Significant differences are indicated as *p* < 0.05 (*), *p* < 0.01 (**), and *p* < 0.001 (***).

Anxiety‐like behavior, assessed in the EPM, was evaluated through the number of entries and time spent in open and closed arms (Figure 3).

Regarding the number of entries into the open arms (Figure 3B), the VPA group showed fewer open‐arm entries compared with the Control group, with an MR VPA/Control = 0.76, 95% CI [0.62–0.92]; *p* = 0.006. Treatment with metformin increased the number of open arm entries compared with the VPA group, with an MR VPA + Metformin/VPA = 1.66, 95% CI [1.40–1.98]; *p* < 0.001. The VPA + Metformin group also showed a higher number of open‐arm entries than the Control group (VPA + Metformin/Control = 1.26, 95% CI [1.04–1.53]; *p* = 0.02).

In sex‐specific simple‐effect analyses, descriptive values are presented in Table 4. In females, the VPA group showed fewer entries into the open arms compared with the Control group (VPA/Control = 0.76, 95% CI [0.61–0.94]; *p* = 0.007) and compared with the VPA + Metformin group (VPA/VPA + Metformin = 0.61, 95% CI [0.49–0.75]; *p* < 0.001). A difference was also observed between the Control and VPA + Metformin groups (Control/VPA + Metformin = 0.80, 95% CI [0.65–0.99]; *p* = 0.032). In males, the VPA group also showed fewer open‐arm entries compared with the Control group (VPA/Control = 0.75, 95% CI [0.61–0.92]; *p* = 0.003) and compared with the VPA + Metformin group (VPA/VPA + Metformin = 0.79, 95% CI [0.64–1.00]; *p* = 0.047), with no difference between the Control and VPA + Metformin groups (Control/VPA + Metformin = 1.06, 95% CI [0.87–1.31]; *p* = 0.753).

A significant group × sex interaction was detected for the number of open‐arm entries (*p* = 0.029), indicating that the response to metformin differed between sexes for this parameter.

For the number of entries into the closed arms (Figure 3A), no difference was observed between VPA and Control groups (VPA/Control = 1.19, 95% CI [0.98–1.45]; *p* = 0.087). However, the VPA + Metformin group showed fewer closed‐arm entries than the VPA group (VPA + Metformin/VPA = 0.79, 95% CI [0.66–0.94]; *p* = 0.008). No difference was observed between the Control and VPA + Metformin groups (VPA + Metformin/Control = 0.94, 95% CI [0.77–1.14]; *p* = 0.519).

In sex‐specific simple‐effect analyses, in females, no differences were observed between Control and VPA groups (Control/VPA = 0.83, 95% CI [0.67–1.03]; *p* = 0.118), whereas the VPA + Metformin group showed fewer closed‐arm entries than the VPA group (VPA + Metformin/VPA = 0.78, 95% CI [0.63–0.97]; *p* = 0.021). No difference was observed between the Control and VPA + Metformin groups (Control/VPA + Metformin = 1.06, 95% CI [0.86–1.31]; *p* = 0.798).

In males, the VPA group showed more closed‐arm entries than the Control group (Control/VPA = 0.77, 95% CI [0.62–0.95]; *p* = 0.009) and then the VPA + Metformin group (VPA + Metformin/VPA = 0.68, 95% CI [0.54–0.85]; *p* < 0.001). No difference was observed between the Control and VPA + Metformin groups (Control/VPA + Metformin = 1.14, 95% CI [0.92–1.40]; *p* = 0.315).

Regarding the time spent in the open arms (Figure 3D), no difference was observed between VPA and Control groups (VPA/Control = 0.87, 95% CI [0.74–1.02]; *p* = 0.081). However, metformin treatment increased the time spent in the open arms compared with the VPA group, with an MR VPA + Metformin/VPA = 1.19, 95% CI [1.01–1.40]; *p* = 0.028. No difference was observed between Control and VPA + Metformin groups (VPA + Metformin/Control = 1.03, 95% CI [0.89–1.20]; *p* = 0.684).

In sex‐specific simple‐effect analyses, in females, no differences were observed between groups (*p* > 0.05). In males, the VPA group showed less time spent in the open arms compared with the Control group (VPA/Control = 0.78, 95% CI [0.65–0.93]; *p* = 0.003) and compared with the VPA + Metformin group (VPA/VPA + Metformin = 0.77, 95% CI [0.63–0.94]; *p* = 0.006), with no difference between Control and VPA + Metformin (Control/VPA + Metformin = 0.99, 95% CI [0.83–1.18]; *p* = 0.989).

For the time spent in the closed arms (Figure 3C), no difference was observed between the VPA and Control groups (VPA/Control = 1.08, 95% CI [0.83–1.41]; *p* = 0.552). However, metformin treatment reduced the time spent in the closed arms compared with the VPA group, with an MR VPA + Metformin/VPA = 0.84, 95% CI [0.71–0.99]; *p* = 0.036. No difference was observed between the Control and VPA + Metformin groups (VPA + Metformin/Control = 0.91, 95% CI [0.70–1.17]; *p* = 0.455).

In sex‐specific simple‐effect analyses, no differences were observed in females (*p* > 0.05). In males, the VPA group spent more time in the closed arms compared with the Control group (Control/VPA = 0.74, 95% CI [0.57–0.95]; *p* = 0.012). No difference was observed between the VPA and VPA + Metformin groups (VPA + Metformin/VPA = 0.86, 95% CI [0.69–1.08]; *p* = 0.258) or between the Control and VPA + Metformin groups (Control/VPA + Metformin = 0.86, 95% CI [0.67–1.10]; *p* = 0.311).

**FIGURE 4:**
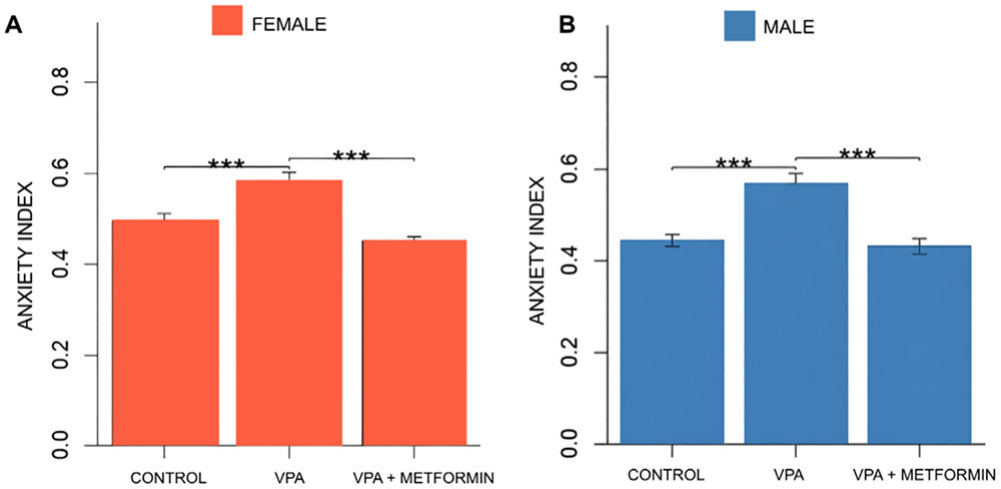
Anxiety index in female and male mice prenatally exposed to VPA and treated with metformin: anxiety index values are shown for (A) females and (B) males in the Control, VPA, and VPA + Metformin groups. Group sizes were females—Control (*n* = 10), VPA (*n* = 10), and VPA + Metformin (*n* = 11); males—Control (*n* = 14), VPA (*n* = 9), and VPA + Metformin (*n* = 9). Data are presented as mean ± SEM. The anxiety index was analyzed using a linear mixed‐effects model, with experimental group, sex, and the group × sex interaction included as fixed effects and litter included as a random effect. Post hoc pairwise comparisons were performed using estimated marginal means with Tukey adjustment for multiple comparisons. Higher anxiety index values indicate greater anxiety‐like behavior. VPA, valproic acid.

The anxiety index was additionally analyzed as an integrated measure of anxiety‐like behavior (Figure 4). A significant effect of experimental group was observed (*F*(2,48) = 32.97; *p* < 0.0001), as well as a significant effect of sex (*F*(1,48) = 4.87; *p* = 0.0321). However, the group × sex interaction was not significant (*F*(2,48) = 0.84; *p* = 0.4380).

In males (Figure 4B), VPA‐exposed animals showed a higher anxiety index than Controls (MD = 0.124, 95% CI [0.066–0.183]; *p* < 0.0001), whereas metformin‐treated animals showed a lower anxiety index than the VPA group (MD = −0.137, 95% CI [−0.197 to −0.078]; *p* < 0.0001). No difference was observed between the Control and VPA + Metformin groups (MD = 0.013, 95% CI [−0.035 to 0.061]; *p* = 0.7937). In females (Figure 4A), the VPA group also showed a higher anxiety index than the Control group (MD = 0.087, 95% CI [0.026–0.147]; *p* = 0.0031), whereas metformin‐treated females showed a lower anxiety index than the VPA group (MD = −0.132, 95% CI [−0.188 to −0.077]; *p* < 0.0001). No significant difference was observed between the Control and VPA + Metformin groups (MD = 0.045, 95% CI [−0.005 to 0.096]; *p* = 0.0846).

### 3.8 Evaluation of Sociability—Three‐Chamber Social Interaction Test

**FIGURE 5:**
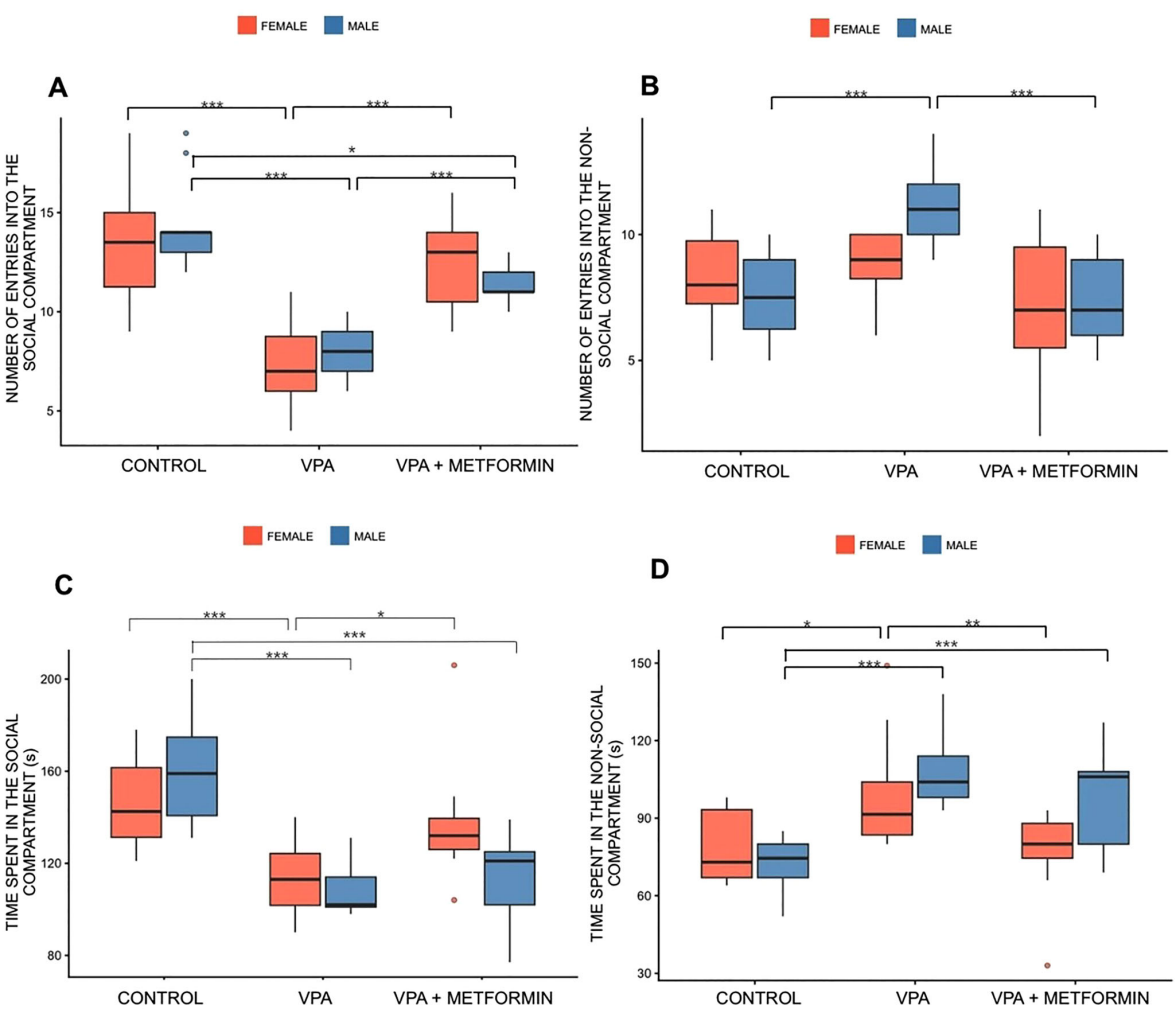
Three‐chamber social interaction performance in female and male mice prenatally exposed to VPA and treated with metformin: (A) number of entries into the compartment with the social stimulus, (B) number of entries into the compartment without the social stimulus, (C) time spent in the compartment with the social stimulus (s), and (D) time spent in the compartment without the social stimulus (s) in the Control, VPA, and VPA + Metformin groups. Group sizes were females—Control (*n* = 10), VPA (*n* = 10), and VPA + Metformin (*n* = 11); males—Control (*n* = 14), VPA (*n* = 9), and VPA + Metformin (*n* = 9). Data are shown as boxplots, with the central line representing the median, boxes indicating the interquartile range, whiskers representing data dispersion, and dots indicating outliers. Statistical analyses were performed using generalized linear mixed models (GLMMs) with a Gamma distribution, with litter included as a random effect. VPA, valproic acid. Significant differences are indicated as *p* < 0.05 (*), *p* < 0.01 (**), and *p* < 0.001 (***).

Social interaction was assessed by the number of entries and time spent in the compartments with and without a social stimulus (Figure 5).

Regarding the number of entries into the compartment with the social stimulus (Figure 5A), the VPA group showed fewer entries into the compartment with the social stimulus compared with the Control group, with an MR VPA/Control = 0.53, 95% CI [0.44–0.65]; *p* < 0.001. Treatment with metformin increased the number of entries into the compartment with the social stimulus compared with the VPA group, with an MR VPA + Metformin/VPA = 1.74, 95% CI [1.47–2.06]; *p* < 0.001. No difference was observed between the VPA + Metformin and Control groups (VPA + Metformin/Control = 0.93, 95% CI [0.77–1.12]; *p* = 0.449).

In sex‐specific simple‐effect analyses, descriptive values are presented in Table 4. In females, the VPA group showed fewer entries into the compartment with the social stimulus compared with the Control group (VPA/Control = 0.53, 95% CI [0.43–0.66]; *p* < 0.001) and compared with the VPA + Metformin group (VPA/VPA + Metformin = 0.58, 95% CI [0.48–0.71]; *p* < 0.001), with no difference between Control and VPA + Metformin groups (Control/VPA + Metformin = 1.09, 95% CI [0.89–1.34]; *p* = 0.563). In males, a similar pattern was observed, with fewer entries into the compartment with the social stimulus in the VPA group compared with Control (VPA/Control = 0.58, 95% CI [0.47–0.70]; *p* < 0.001) and VPA + Metformin (VPA/VPA + Metformin = 0.72, 95% CI [0.57–0.89]; *p* < 0.001). Additionally, a difference was observed between Control and VPA + Metformin groups (Control/VPA + Metformin = 1.24, 95% CI [1.02–1.51]; *p* = 0.027).

For the number of entries into the compartment without the social stimulus (Figure 5B), no difference was observed between VPA and Control groups (VPA/Control = 1.08, 95% CI [0.82–1.43]; *p* = 0.575). However, the VPA + Metformin group showed fewer entries into the compartment without the social stimulus than the VPA group (VPA + Metformin/VPA = 0.79, 95% CI [0.63–0.99]; *p* = 0.040). No difference was observed between the Control and VPA + Metformin groups (VPA + Metformin/Control = 0.86, 95% CI [0.65–1.12]; *p* = 0.262).

In sex‐specific simple‐effect analyses, males in the VPA group showed more entries into the compartment without the social stimulus compared with Control (Control/VPA = 0.67, 95% CI [0.52–0.88]; *p* = 0.002) and compared with the VPA + Metformin group (VPA + Metformin/VPA = 0.64, 95% CI [0.48–0.87]; *p* = 0.002), with no difference between Control and VPA + Metformin (Control/VPA + Metformin = 1.05, 95% CI [0.80–1.37]; *p* = 0.912). No differences were observed in females.

Regarding the time spent in the compartment with the social stimulus (Figure 5C), the VPA group showed less time spent in the compartment with the social stimulus compared with the Control group, with an MR VPA/Control = 0.80, 95% CI [0.69–0.93]; *p* = 0.004. Treatment with metformin increased the time spent in the compartment with the social stimulus compared with the VPA group, with an MR VPA + Metformin/VPA = 1.17, 95% CI [1.04–1.32]; *p* = 0.007, with no difference between VPA + Metformin and Control groups (*p* = 0.448).

In sex‐specific simple‐effect analyses, in females, the VPA group showed less time spent in the compartment with the social stimulus compared with Control (VPA/Control = 0.79, 95% CI [0.68–0.92]; *p* < 0.001) and compared with the VPA + Metformin group (VPA/VPA + Metformin = 0.84, 95% CI [0.72–0.97]; *p* = 0.013), with no difference between Control and VPA + Metformin (Control/VPA + Metformin = 1.06, 95% CI [0.92–1.23]; *p* = 0.597). In males, the VPA group also showed less time spent in the compartment with the social stimulus compared with Control (VPA/Control = 0.67, 95% CI [0.58–0.78]; *p* < 0.001). No difference was observed between the VPA and VPA + Metformin groups (VPA/VPA + Metformin = 0.93, 95% CI [0.80–1.10]; *p* = 0.580), whereas the Control group showed more time in the social‐stimulus compartment than the VPA + Metformin group (Control/VPA + Metformin = 1.39, 95% CI [1.20–1.61]; *p* < 0.001).

For the time spent in the compartment without the social stimulus (Figure 5D), the VPA group showed more time spent in the compartment without the social stimulus compared with the Control group, with an MR VPA/Control = 1.24, 95% CI [1.00–1.52]; *p* = 0.045. Treatment with metformin reduced the time spent in the compartment without the social stimulus compared with the VPA group, with an MR VPA + Metformin/VPA = 0.80, 95% CI [0.68–0.93]; *p* = 0.004. No difference was observed between the Control and VPA + Metformin groups (*p* = 0.869).

In females, the VPA group spent more time in the nonsocial‐stimulus compartment than the Control group (Control/VPA = 0.80, 95% CI [0.65–0.98]; *p* = 0.028) and than the VPA + Metformin group (VPA + Metformin/VPA = 0.78, 95% CI [0.64–0.95]; *p* = 0.009), with no difference between the Control and VPA + Metformin groups (Control/VPA + Metformin = 1.02, 95% CI [0.84–1.24]; *p* = 0.966).

In males, the VPA group also showed more time spent in the compartment without the social stimulus compared with Control (VPA/Control = 1.53, 95% CI [1.25–1.85]; *p* < 0.001), with no difference between VPA and VPA + Metformin (*p* = 0.379), whereas the VPA + Metformin group also spent more time in the compartment without the social stimulus than the Control group (Control/VPA + Metformin = 0.74, 95% CI [0.61–0.90]; *p* < 0.001). A significant group × sex interaction was also observed for time spent in the nonsocial‐stimulus compartment (*p* = 0.004), supporting a sex‐dependent pattern for this outcome.

**FIGURE 6:**
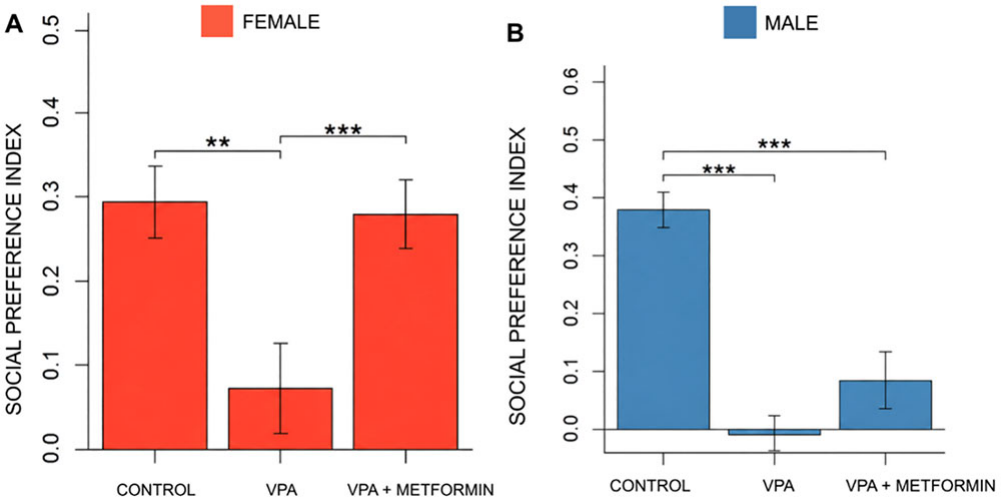
Social preference index (SPI) in female and male mice prenatally exposed to VPA and treated with metformin. SPI values are shown for (A) females and (B) males in the Control, VPA, and VPA + Metformin groups. Group sizes were females—Control (*n* = 10), VPA (*n* = 10), and VPA + Metformin (*n* = 11); males—Control (*n* = 14), VPA (*n* = 9), and VPA + Metformin (*n* = 9). Data are presented as mean ± SEM. The SPI was analyzed using a linear mixed‐effects model, with experimental group, sex, and the group × sex interaction included as fixed effects, litter included as a random effect, and heterogeneity of residual variance among experimental groups accounted for in the model. Post hoc pairwise comparisons were performed using estimated marginal means with Tukey adjustment for multiple comparisons. VPA, valproic acid.      .

The SPI was additionally analyzed as an integrated measure of social preference (Figure 6). A significant effect of experimental group was observed (*F*(2,48) = 15.92; *p* < 0.0001), whereas the effect of sex was not significant (*F*(1,48) = 1.40; *p* = 0.243). A significant group × sex interaction was detected (*F*(2,48) = 7.55; *p* = 0.0014), indicating that the effect of experimental group on social preference differed between males and females.

In females (Figure 6A), the VPA group showed a lower SPI than the Control group (MD = −0.203, 95% CI [−0.363 to −0.042]; *p* = 0.010) and the VPA + Metformin group (MD = −0.188, 95% CI [−0.323 to −0.053]; *p* = 0.0042). No difference was observed between the Control and VPA + Metformin groups (MD = 0.015, 95% CI [−0.137 to 0.167]; *p* = 0.969).

In males (Figure 6B), the VPA group showed a lower SPI than the Control group (MD = −0.383, 95% CI [−0.542 to −0.224]; *p* < 0.0001). The VPA + Metformin group also showed a lower SPI than the Control group (MD = −0.297, 95% CI [−0.449 to −0.144]; *p* = 0.0001), whereas no significant difference was observed between the VPA and VPA + Metformin groups (MD = −0.086, 95% CI [−0.231 to 0.058]; *p* = 0.326).

Comparisons between sexes within each experimental group showed a significant difference in the VPA + Metformin group, with females exhibiting higher SPI values than males (MD = 0.178, 95% CI [0.068–0.288]; *p* = 0.0021). Control males showed higher SPI values than Control females (MD = 0.104, 95% CI [0.004–0.203]; *p* = 0.0418), whereas no significant sex difference was detected in the VPA group (Male—Female MD = −0.077, 95% CI [−0.198 to 0.045]; *p* = 0.210).

### 3.9 Evaluation of Working Memory—Y‐Maze Test

**FIGURE 7:**
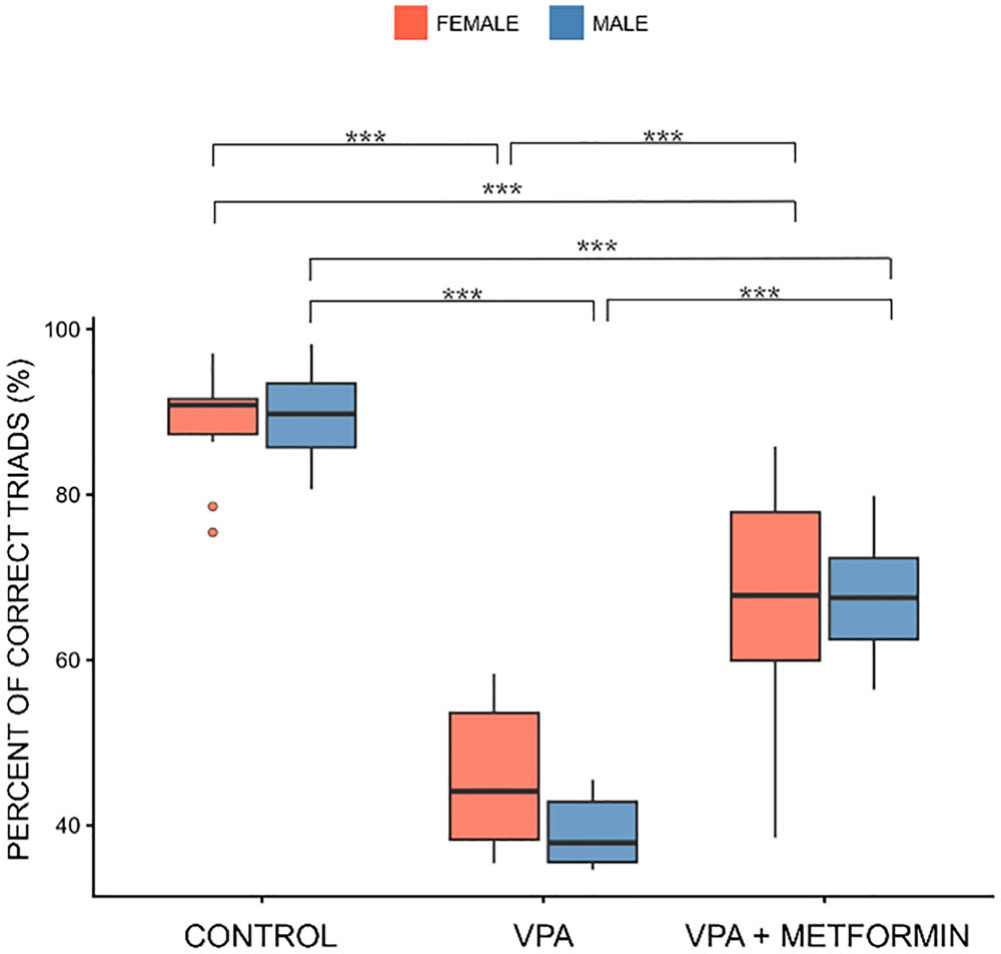
Spatial working memory performance in female and male mice prenatally exposed to VPA and treated with metformin. Percentage of correct triads in the Y‐maze test for the Control, VPA, and VPA + Metformin groups. Group sizes were females—Control (*n* = 10), VPA (*n* = 10), and VPA + Metformin (*n* = 11); males—Control (*n* = 14), VPA (*n* = 9), and VPA + Metformin (*n* = 9). Data are shown as boxplots, with the central line representing the median, boxes indicating the interquartile range, whiskers representing data dispersion, and dots indicating outliers. Statistical analyses were performed using generalized linear mixed models (GLMMs) with a Gamma distribution, with litter included as a random effect. VPA, valproic acid.

Spatial working memory, assessed by the percentage of correct triads in the Y‐maze test, was reduced in animals exposed to VPA compared with the Control group (Figure 7). In the overall analysis, the VPA group showed a marked reduction in performance, with an MR VPA/Control = 0.51, 95% CI [0.45–0.57]; *p* < 0.001, indicating a lower percentage of correct triads.

Treatment with metformin improved spatial working memory performance compared with the VPA group, with an MR VPA + Metformin/VPA = 1.48, 95% CI [1.33–1.66]; *p* < 0.001. However, the VPA + Metformin group remained significantly lower than the Control group (VPA + Metformin/Control = 0.76, 95% CI [0.67–0.85]; *p* < 0.001).

In sex‐specific simple‐effect analyses, descriptive values (mean ± SD) for each experimental group are presented in Table 4. In females, the VPA group showed reduced performance compared with the Control group (VPA/Control = 0.51, 95% CI [0.45–0.59]; *p* < 0.001) and compared with the VPA + Metformin group (VPA/VPA + Metformin = 0.67, 95% CI [0.59–0.78]; *p* < 0.001). The VPA + Metformin group also remained lower than Control (Control/VPA + Metformin = 1.32, 95% CI [1.15–1.51]; *p* < 0.001).

A similar pattern was observed in males, with reduced performance in the VPA group compared with Control (VPA/Control = 0.43, 95% CI [0.38–0.50]; *p* < 0.001) and compared with VPA + Metformin (VPA/VPA + Metformin = 0.57, 95% CI [0.50–0.67]; *p* < 0.001). The VPA + Metformin group also showed lower performance than Control (Control/VPA + Metformin = 1.32, 95% CI [1.16–1.51]; *p* < 0.001).

A significant group × sex interaction was detected for the percentage of correct triads (*p* = 0.048), indicating that the response to metformin differed between sexes for this parameter.

### 3.10 Brain Oxidative Stress, Total Protein Content, and Cholinergic Markers

Biochemical analyses were performed in the PFC and HPC. As shown in Table 5, GSH levels were lower in the VPA group than in the corresponding Control group in the HPC and PFC of both sexes. Compared with the VPA group, metformin was associated with higher GSH levels in the female HPC and male PFC, whereas no significant differences were detected in the female PFC or male HPC. Regarding MDA, an indicator of lipid peroxidation, VPA increased its levels in both brain areas and sexes. Compared with VPA, metformin was associated with lower MDA levels in the female PFC and in both brain regions in males, whereas no significant difference was observed in the female HPC.

**TABLE 5:** Effect of metformin on oxidative stress markers, total protein content, and acetylcholinesterase (AChE) activity in the hippocampus (HPC) and prefrontal cortex (PFC) of female and male mice prenatally exposed to valproic acid (VPA).

	Females				Males		
		Control	VPA	VPA + Metformin	Control	VPA	VPA + Metformin
**GSH**	HPC	634.0 ± 60.1	458.8 ± 38.2^*^	757.9 ± 45.6^##^	861.2 ± 50.6	364.1 ± 55.7^***^	676.4 ± 21.0
	PFC	754.5 ± 62.7	493.7 ± 36.0^**^	602.7 ± 29.8	868.7 ± 52.2	515.3 ± 40.2^**^	810.5 ± 89.12^#^
**MDA**	HPC	260.5 ± 38.8	875.4 ± 195.6^**^	404.5 ± 73.2	348.0 ± 41.9	732.0 ± 23.3^***^	288.4 ± 48.6^###^
	PFC	383.3 ± 50.6	1224 ± 158.9^***^	255.6 ± 54.8^###^	391.7 ± 37.9	733.0 ± 63.4^*^	377.6 ± 80.5^#^
**TP**	HPC	541.3 ± 48.7	462.2 ± 50.2	525.7 ± 74.1	660.9 ± 28.7	468.7 ± 59.2^*^	585.3 ± 13.0
	PFC	533.0 ± 87.6	169.2 ± 18.2^**^	506.6 ± 66.2^#^	165.3 ± 8.9	98.6 ± 6.5^*^	161.0 ± 8.6^#^
**AChE**	HPC	4.2 ± 0.3	6.2 ± 1.5	7.6 ± 3.6	5.4 ± 0.6	11.6 ± 2.2	4.7 ± 0.5^#^
	PFC	30.1 ± 5.7	112.0 ± 15.3^**^	53.8 ± 18.7^#^	20.3 ± 5.8	60.8 ± 5.0^***^	17.9 ± 5.3^###^

*Note*: Data are expressed as mean ± SEM. Statistical analyses were performed using one‐way ANOVA followed by Tukey's post hoc test or the Kruskal–Wallis test followed by Dunn's post hoc test, as appropriate. *p < 0.05, **p < 0.01, ***p < 0.001 vs. the corresponding Control group; ^#^ *p* < 0.05, ^##^ *p* < 0.01, ^###^ *p* < 0.001 vs. the corresponding VPA group. No significant differences were detected between the Control and VPA + Metformin groups.

Abbreviations: AChE, acetylcholinesterase activity (nmol/min/mg protein); GSH, reduced glutathione (µg/mL); MDA, malondialdehyde (nmol/mg tissue); TP, total protein content (µg/mL).

Regarding total protein content, no significant differences were observed among groups in the female HPC. In the female PFC, total protein content was lower in the VPA group than in the Control group, whereas the VPA + Metformin group showed higher values than the VPA group. In males, VPA‐exposed animals showed lower total protein content than Controls in both brain regions; however, a significant difference between the VPA and VPA + Metformin groups was observed in the PFC but not in the HPC.

For AChE, VPA increased its activity in PFC of both sexes. Metformin was associated with lower PFC AChE activity than the VPA group in both sexes. In the HPC, no significant differences were observed among groups in females, whereas metformin‐treated males showed lower AChE activity than the VPA group.

Overall, metformin‐associated changes in GSH, MDA, total protein content, and AChE activity varied according to sex and brain region, with the most consistent effects observed for MDA and PFC AChE activity.

## 4 Discussion

The present study extends previous preclinical evidence on the effects of metformin in ASD‐related animal models (Elnahas et al. 2022; Atia et al. 2023; Wang et al. 2018) by showing that post‐weaning metformin treatment attenuated several behavioral alterations associated with prenatal VPA exposure in male and female mice. These effects involved multiple behavioral domains, including locomotor and vertical exploratory activity, repetitive self‐grooming, anxiety‐like behavior, sociability, and spatial working memory, and were accompanied by changes in redox‐related markers, total protein content, and AChE activity in the PFC and HPC. Importantly, the effects of metformin were not uniform across all behavioral parameters, sexes, or brain regions, and significant group × sex interactions were restricted to specific outcomes. Thus, the present findings support a parameter‐dependent rather than generalized influence of sex on treatment response, consistent with previous evidence that prenatal VPA‐associated phenotypes may differ between male and female animals (Gouda et al. 2022).

### 4.1 Maternal, Litter, and Offspring Survival Outcomes

Prenatal VPA exposure was associated with substantial maternal and litter losses in the present study. Three of the 15 VPA‐exposed dams died following VPA administration, and 6 of the 12 surviving dams subsequently experienced complete litter loss due to cannibalism, whereas no maternal deaths or complete litter losses occurred in the Control group. Consequently, only six VPA‐exposed litters were available for post‐weaning follow‐up. Adverse reproductive and maternal outcomes have also been reported in prenatal VPA models.

Jain et al. (2024), using VPA administration on GD12.5 in pregnant Wistar rats, reported reduced litter size, increased maternal cannibalism, complete pregnancy loss in some dams, and reduced offspring survival. Although differences in species and VPA dose preclude direct quantitative comparison with the present findings, these observations indicate that substantial litter loss and disruption of maternal behavior can occur following prenatal VPA exposure. Importantly, because the original number of offspring in the completely lost litters could not be determined in the present study, litter‐size data are restricted to the surviving litters and should not be interpreted as an estimate of the overall effect of VPA on litter size.

Post‐weaning survival also differed descriptively among experimental groups. All Control offspring survived the 15‐day treatment period, whereas survival was 82.6% in VPA‐exposed offspring receiving vehicle and 90.9% in VPA‐exposed offspring treated with metformin. However, these differences were not subjected to inferential survival analysis and, therefore, should not be interpreted as evidence of a protective effect of metformin on offspring survival.

Previous studies have shown that developmental outcomes, following prenatal VPA exposure, can vary substantially according to dose and experimental protocol, with reports ranging from normal postnatal survival to increased fetal loss and other adverse developmental outcomes (Jain et al. 2024; Favre et al. 2013). In the present study, the differential attrition observed among VPA‐exposed offspring may have influenced the composition of the cohort available for subsequent behavioral and biochemical analyses and should, therefore, be considered when interpreting these outcomes.

Mean body weight increased from D1 to D15 in all experimental groups and in both sexes. However, because only group‐level mean values were retained, individual variability and longitudinal treatment effects could not be statistically evaluated. Therefore, the apparent differences in body‐weight gain among the Control, VPA, and VPA + Metformin groups should be interpreted descriptively and should not be considered evidence of either impaired growth following prenatal VPA exposure or an effect of metformin on postnatal weight gain. This cautious interpretation is also consistent with previous evidence showing that prenatal VPA exposure does not necessarily produce persistent alterations in offspring body mass. Favre et al. (2013), for example, reported developmentally similar body‐mass gain between control and VPA‐exposed offspring despite other adverse developmental outcomes in the VPA model.

### 4.2 Behavioral Effects of Metformin in VPA‐Exposed Offspring

Prenatal VPA exposure altered spontaneous activity, with reduced locomotor activity and increased vertical exploration, and also increased repetitive self‐grooming. Metformin attenuated these alterations, increasing crossings and reducing rearing and grooming relative to untreated VPA‐exposed offspring. Although changes in open‐field activity are not consistently reproduced across prenatal VPA protocols, increased repetitive behavior is a recurrent feature of ASD‐related models. Previous studies have similarly shown that metformin attenuates repetitive and other behavioral abnormalities in both VPA‐exposed animals and BTBR mice (Ishola et al. 2020; Atia et al. 2023; Wang et al. 2018). Notably, the effect observed here was more consistent for grooming frequency than duration, reinforcing that the behavioral response to both VPA and metformin may depend on the specific parameter evaluated rather than reflecting a uniform change in activity.

Metformin also attenuated alterations in emotional and social behavior, although these effects varied across parameters and sexes. In the EPM, individual measures of entries into and time spent in the open and closed arms showed a heterogeneous pattern, whereas the integrated anxiety index consistently indicated greater anxiety‐like behavior after prenatal VPA exposure and lower values following metformin treatment in both sexes. In contrast, social behavior revealed a clearer sex‐dependent response. Prenatal VPA reduced social preference, but metformin increased the SPI relative to VPA in females, whereas no significant improvement was detected in males. This group × sex interaction indicates that the effect of metformin on social preference cannot be generalized across sexes. Such heterogeneity is consistent with previous evidence showing sex‐related differences in anxiety‐like and social phenotypes following prenatal VPA exposure (Scheggi et al. 2020; Gouda et al. 2022; Mamali et al. 2025), emphasizing the importance of evaluating male and female offspring within the same experimental design.

Prenatal VPA exposure also impaired spatial working‐memory performance in the Y‐maze, whereas metformin increased the percentage of correct triads in both sexes. However, metformin‐treated animals remained significantly different from Controls, indicating attenuation rather than complete reversal of the cognitive deficit. A significant group × sex interaction further indicated that the magnitude of this response differed between males and females. Together with the sex‐related effects observed for social preference and open‐arm entries, this finding suggests that sex selectively modulated metformin responsiveness across behavioral domains rather than exerting a uniform effect. Sex‐related differences in cognitive and social phenotypes have also been described following prenatal VPA exposure (Scheggi et al. 2020; Gouda et al. 2022; Ghahremani et al. 2022), reinforcing the importance of including both sexes when evaluating pharmacological interventions in this model.

These behavioral domains should not be considered entirely independent because changes in locomotor/exploratory activity or anxiety‐like behavior may influence performance in social and cognitive tasks; therefore, individual test outcomes should not be interpreted as isolated ASD‐specific deficits.

The behavioral effects of metformin may be interpreted within a broader neurobiological context involving redox homeostasis and cholinergic signaling. Oxidative imbalance can disrupt neuronal and synaptic function, whereas cholinergic transmission contributes to cognitive, exploratory, and repetitive behavioral processes. Importantly, previous VPA studies have reported behavioral improvement in parallel with attenuation of oxidative alterations and abnormal AChE activity, including after metformin treatment (Eissa et al. 2020; Ishola et al. 2020). Experimental modulation of cholinergic signaling has also been shown to modify repetitive behavior in ASD‐related models (Amodeo et al. 2014). These findings provide biological plausibility for the parallel behavioral, redox, and cholinergic changes observed in the present study; however, because brain–behavior correlations and direct mechanistic manipulations were not performed, these pathways cannot be considered demonstrated mediators of the behavioral effects of metformin.

### 4.3 Biochemical Effects of Metformin

Consistent with this neurobiological context, the biochemical findings indicated that metformin also modulated redox‐related alterations associated with prenatal VPA exposure. Changes in GSH and MDA were not uniform across the PFC and HPC or between sexes, indicating a region‐ and sex‐dependent redox response to treatment. This pattern is relevant because GSH reflects endogenous antioxidant capacity, whereas MDA provides an index of lipid peroxidation, allowing complementary aspects of redox homeostasis to be assessed. Previous work in the prenatal VPA model similarly showed that postnatal metformin treatment improved antioxidant defenses and reduced indices of oxidative damage (Ishola et al. 2020). Thus, the present findings support attenuation of selected components of VPA‐associated oxidative imbalance rather than a generalized normalization of redox status. Although activation of antioxidant pathways such as AMPK/Nrf2 has been demonstrated among the neuroprotective actions of metformin under experimental oxidative stress (Yang et al. 2020), these pathways were not assessed here and cannot be directly implicated in the present effects.

Closer examination of the redox markers revealed that the metformin‐associated response differed according to both the marker and brain region examined. GSH was significantly increased relative to VPA in the female HPC and male PFC, whereas no significant treatment‐related increase was detected in the female PFC or male HPC. MDA showed a partly distinct pattern, with significant reductions after metformin treatment in the female PFC and in both brain regions of males, but not in the female HPC. These findings indicate that enhancement of antioxidant capacity and reduction of lipid peroxidation did not necessarily occur in parallel within the same region. Such dissociation is biologically plausible because GSH availability and lipid peroxidation reflect related but distinct components of redox homeostasis. Moreover, recent evidence from prenatal VPA models has demonstrated sex‐ and region‐specific neurochemical vulnerability, including selective reductions in hippocampal GSH in female offspring (Martins et al. 2026). Thus, the present findings suggest that the redox response associated with metformin is heterogeneous across neural regions and sexes rather than uniformly antioxidant throughout the brain.

Total protein content showed a comparatively limited and region‐specific response. Prenatal VPA exposure was associated with lower protein levels in selected regions, whereas metformin‐related increases relative to VPA were mainly observed in the PFC of both sexes. Because the Bradford assay provides a global estimate of tissue protein content, these changes cannot be attributed to specific structural, synaptic, or signaling proteins. Although metformin has been reported to influence neuronal protein expression under experimental stress conditions (Yang et al. 2023), the present findings should therefore be interpreted as a nonspecific biochemical alteration rather than evidence of restored protein synthesis or neuronal integrity.

AChE activity showed a more consistent treatment‐related pattern in the PFC, where prenatal VPA exposure increased enzyme activity in both sexes and metformin attenuated this increase. Because AChE is responsible for acetylcholine hydrolysis, increased enzymatic activity may reflect altered cholinergic regulation, although AChE activity alone does not directly indicate synaptic acetylcholine availability. Similar increases in AChE activity in the PFC and HPC have been reported after prenatal VPA exposure, with postnatal metformin attenuating these alterations (Ishola et al. 2020). Cholinergic dysfunction is particularly relevant to the behavioral domains evaluated here, as experimental enhancement of acetylcholine signaling has been shown to reduce cognitive rigidity and improve social behavior in ASD‐related mouse models (Karvat and Kimchi 2014), whereas pharmacological modulation of AChE has also been associated with attenuation of repetitive and anxiety‐like behaviors in VPA‐exposed mice (Eissa et al. 2020). Thus, the reduction in PFC AChE activity after metformin represents a parallel neurochemical finding that is biologically relevant to the behavioral domains examined, but a causal relationship cannot be established.

Overall, the biochemical findings indicate that metformin‐associated changes were not uniform across markers or brain regions. Redox responses varied between the PFC and HPC and showed distinct patterns in males and females, whereas the attenuation of increased AChE activity was more consistently observed in the PFC of both sexes. This heterogeneity suggests that different neurochemical systems and brain regions may differ in their sensitivity to post‐weaning metformin treatment following prenatal VPA exposure. Similar regional variability in oxidative and cholinergic alterations has been reported in VPA models (Eissa et al. 2020; Ishola et al. 2020; Martins et al. 2026). Importantly, because biochemical analyses were performed separately within each sex rather than through a formal group × sex interaction model, these findings should be interpreted as sex‐specific patterns rather than definitive evidence of sex‐dependent biochemical treatment effects.

### 4.4 Limitations and Conclusion

Several limitations should be considered when interpreting these findings. Only a single metformin dose and post‐weaning treatment window were evaluated, limiting conclusions regarding dose–response relationships, optimal treatment timing, and persistence of the observed effects. In addition, the absence of a Control + Metformin group prevents determining whether metformin produces behavioral or biochemical effects independently of prenatal VPA exposure. Repeated oral gavage may also represent a source of procedural stress; however, the same administration schedule was applied across experimental groups. Although redox‐related markers and AChE activity were assessed, the oxidative stress evaluation was restricted to GSH and MDA and did not include first‐line antioxidant enzymes such as SOD and CAT. Furthermore, inflammatory mediators, synaptic proteins, and intracellular signaling pathways such as AMPK/mTOR or Nrf2 were not directly examined, and no brain–behavior correlation analysis was performed. Future studies incorporating histological and molecular markers, including GFAP, Iba1, NeuN, PSD95, synaptophysin, BDNF, and phosphorylated AMPK/mTOR, could provide important mechanistic validation of the observed effects. Therefore, the behavioral and biochemical findings should be regarded as parallel observations with biological plausibility rather than evidence of a demonstrated mechanistic relationship.

A further limitation relates to attrition throughout the experimental protocol. Substantial maternal and complete litter losses occurred after prenatal VPA exposure, followed by additional mortality during the post‐weaning treatment period, so the animals available for behavioral and biochemical analyses necessarily represented the surviving cohort. Although multiple independent litters contributed to each experimental group, a potential survivor‐related selection effect cannot be excluded. Developmental progression was not systematically assessed beyond body‐weight and survival monitoring, and body‐weight data were available only as group‐level means, precluding longitudinal inferential analysis. In addition, biochemical comparisons were performed separately within each sex rather than through a formal group × sex interaction model. Consequently, differences observed between males and females in biochemical responses should be interpreted as sex‐specific patterns rather than definitive evidence of sex‐dependent treatment effects.

In conclusion, post‐weaning metformin treatment attenuated several behavioral alterations associated with prenatal VPA exposure and was accompanied by changes in redox‐related markers and AChE activity in the PFC and HPC. Importantly, these effects were not uniform across behavioral domains, brain regions, or sexes, highlighting the relevance of considering biological sex when evaluating pharmacological responses in preclinical ASD models. The parallel behavioral and biochemical findings support further investigation of metformin in this context, particularly to determine the molecular pathways underlying these effects and whether they persist beyond the treatment period.

## Author Contributions

**Madna Costa Freitas**, **Ana Maria Sampaio Assreuy**, and **Gislei Frota Aragão** contributed to the experimental design. **Madna Costa Freitas** participated in the mating, induction, treatment, behavioral testing, and biochemical analysis procedures. **Iara Kessila Milhome Vasconcelos**, **Renê Felipe de Freitas**, **David Ribeiro Fontenele**, **Pedro Otávio de Freitas Alves**, **Victoria Soares Diógenes**, **Isadora Porto de Andrade**, and **Maria Helena da Silva Pitombeira** participated in the treatment, behavioral testing, and biochemical analysis procedures. **Madna Costa Freitas** performed data analysis and contributed to manuscript writing and revision. **Ana Maria Sampaio Assreuy** and **Gislei Frota Aragão** contributed to manuscript writing and revision. All authors read and approved the final version of the manuscript.

## Funding

The authors have nothing to report.

## Ethics Statement

The experimental protocol and procedures were reviewed and approved by the Animal Research Ethics Committee of the State University of Ceará (CEUA/UECE, protocol number NUP 31032.000519/2023‐49). All animal experiments were conducted in accordance with the Brazilian guidelines for the care and use of laboratory animals (CONCEA) and following the ARRIVE guidelines for the reporting of animal research.

## Conflicts of Interest

The authors declare no conflicts of interest.

## Data Availability

The datasets generated and/or analyzed during the present study are available from the corresponding author upon request.
